# Supplementary material for: State-transition dynamics of resting-state functional magnetic resonance imaging data: model comparison and test-to-retest analysis
Source: BMC Neurosci. 2024 Mar 4;25:14. doi: 10.1186/s12868-024-00854-3 (PMC10913599; doi:10.1186/s12868-024-00854-3)
Supplement: Supplementary file 1 — Additional file 1. Supplementary information. [file 12868_2024_854_MOESM1_ESM.pdf]

Supplementary Information for
State-transition dynamics of resting-state functional magnetic resonance imaging data: Model comparison and test-to-retest analysis

Saiful Islam, Pitambar Khanra, Johan Nakuci, Sarah F. Muldoon, Takamitsu Watanabe, Naoki Masuda

S1. Correspondence between each AAL ROI and the brain system

As we described in section “Human Connectome Project data”, we mapped the 116 ROIs of the AAL atlas for the HCP data to different representative brain systems based on the Euclidian distance between the centroid of each ROI in the AAL atlas and the centroid of each ROI in the Schaefer atlas. Then, we excluded 42 ROIs labeled as ‘subcortical’ or ‘cerebellar’. With this exclusion, we consider the remaining seven brain systems: the control network, default mode network (DMN), dorsal attention network (DAN), limbic network, salience/ventral attention network, somatomotor network, and the visual network. We show the mapping between these brain systems and the AAL ROIs in Table S1.

Table S1: Mapping of AAL ROIs to the brain system

Brain system	ROI in the AAL atlas
Control network	8, 10, 13, 14, 32, 34, 61, 66, 88, 89
DMN	3, 4, 9, 15, 16, 23, 24, 25, 26, 31, 33, 65, 68, 82, 83, 84, 85, 86, 90
DAN	11, 12, 57, 59, 60, 62, 67, 69, 70
Limbic network	5, 6, 19, 20, 27, 28, 87
Salience/ventral attention network	1, 7, 29, 30, 63, 64
Somatomotor network	2, 17, 18, 45, 46, 49, 50, 58, 79, 80, 81
Visual network	35, 36, 43, 44, 47, 48, 51, 52, 53, 54, 55, 56

S2. Quality of clustering without global signal removal

We evaluate the quality of clustering when the global signal removal is omitted as follows. We consider the DMN extracted from the MSC, which has $\tilde{N} = 12$ ROIs, and the symmetrized variant of the DMN, which has $\tilde{N} = 8$ ROIs and is obtained by averaging of the time series over each pair of symmetric ROIs from the left and right hemispheres. The non-symmetrized DMN corresponds to the third treatment described in the main text. The symmetrized DMN corresponds to the first and second treatments. In this section, we use fMRI signals from these DMNs without global signal removal.

As we did in Fig. 1 in the main text, we ran the seven clustering methods for each number of clusters, $K \in \{2, \cdots, 10\}$, each of the ten sessions, and each of the eight participants. Then, we calculated the $\text{GEV}_{\text{total}}$ and the WCSS. We show the $\text{GEV}_{\text{total}}$ values in Figs. S1(a)–(b) and the WCSS values in Figs. S1(c)–(d). Figures S1(a) and (c) correspond to the 8-ROI DMN, and Figs. S1(b) and (d) correspond to the 12-ROI DMN. For the 8-ROI DMN, we find that $\text{GEV}_{\text{total}}$ is smaller without global signal removal (shown in Fig. S1(a)) than with global signal removal (shown in Figs. 1(a) and (b) in the main text). Moreover, the WCSS is notably larger without global signal removal (shown in Fig. S1(c)) than with global signal removal (shown in Figs. 1(d) and (e)). These results are qualitatively the same for the 12-ROI DMN (see Figs. S1(b) and (d), which should be compared with Figs. 1(c) and (f), respectively). Therefore, for the present data, the global signal removal improves the clustering analysis.

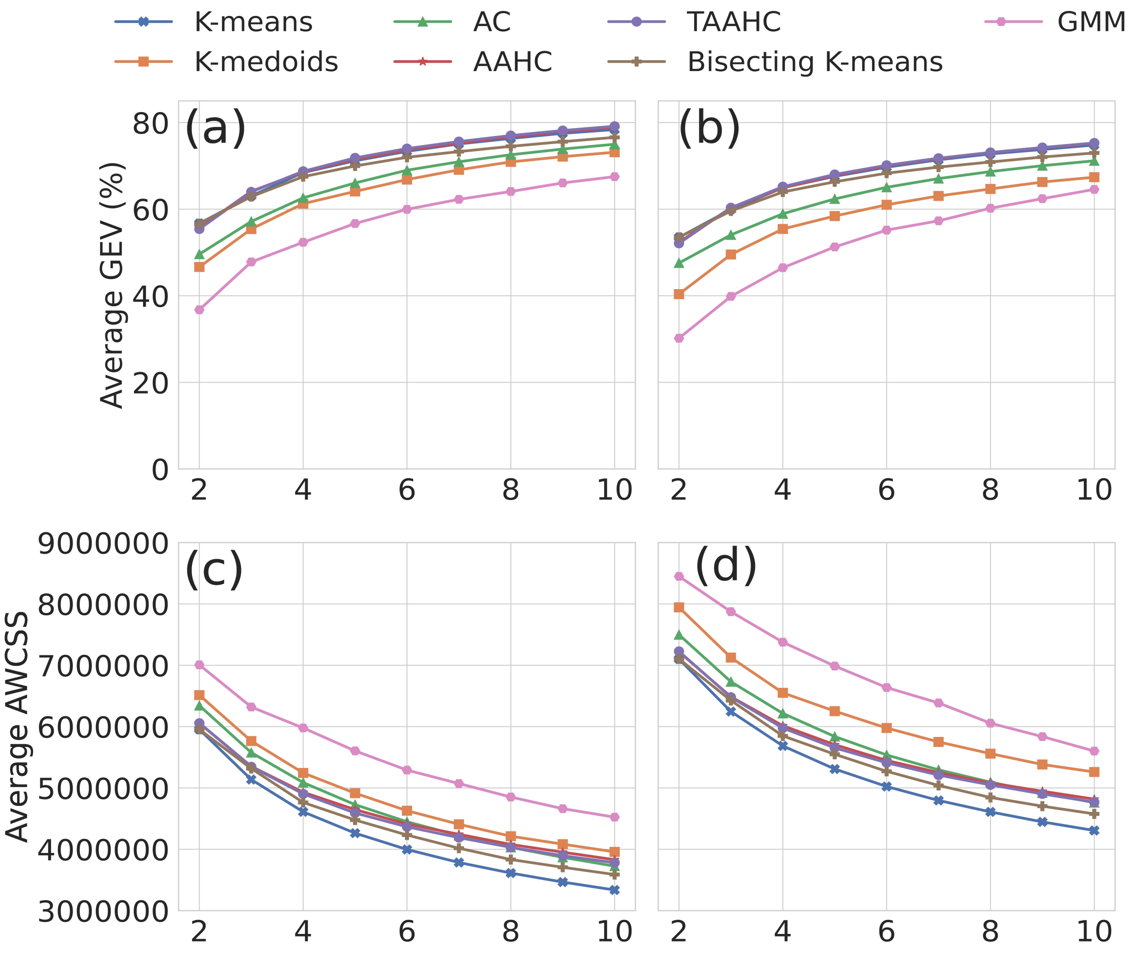

Figure S1: Performance of estimating discrete states without global signal removal. We use the DMN extracted from the MSC data. We show the results for the seven clustering methods and $K \in \{2, \dots, 10\}$. (a) $\text{GEV}_{\text{total}}$, $\tilde{N} = 8$ ROIs. (b) $\text{GEV}_{\text{total}}$, $\tilde{N} = 12$. (c) WCSS, $\tilde{N} = 8$. (d) WCSS, $\tilde{N} = 12$. Each $\text{GEV}_{\text{total}}$ and WCSS value shown is the average over the eight participants and ten sessions per participant.

S3. Within-participant and between-participant reproducibility of the state-transition dynamics with $K = 7$ and $K = 10$

We show the distributions of the discrepancy in terms of the five observables for the within-participant and between-participant comparisons in Figs. S2 and S3 for the number of clusters $K = 7$ and $K = 10$, respectively.

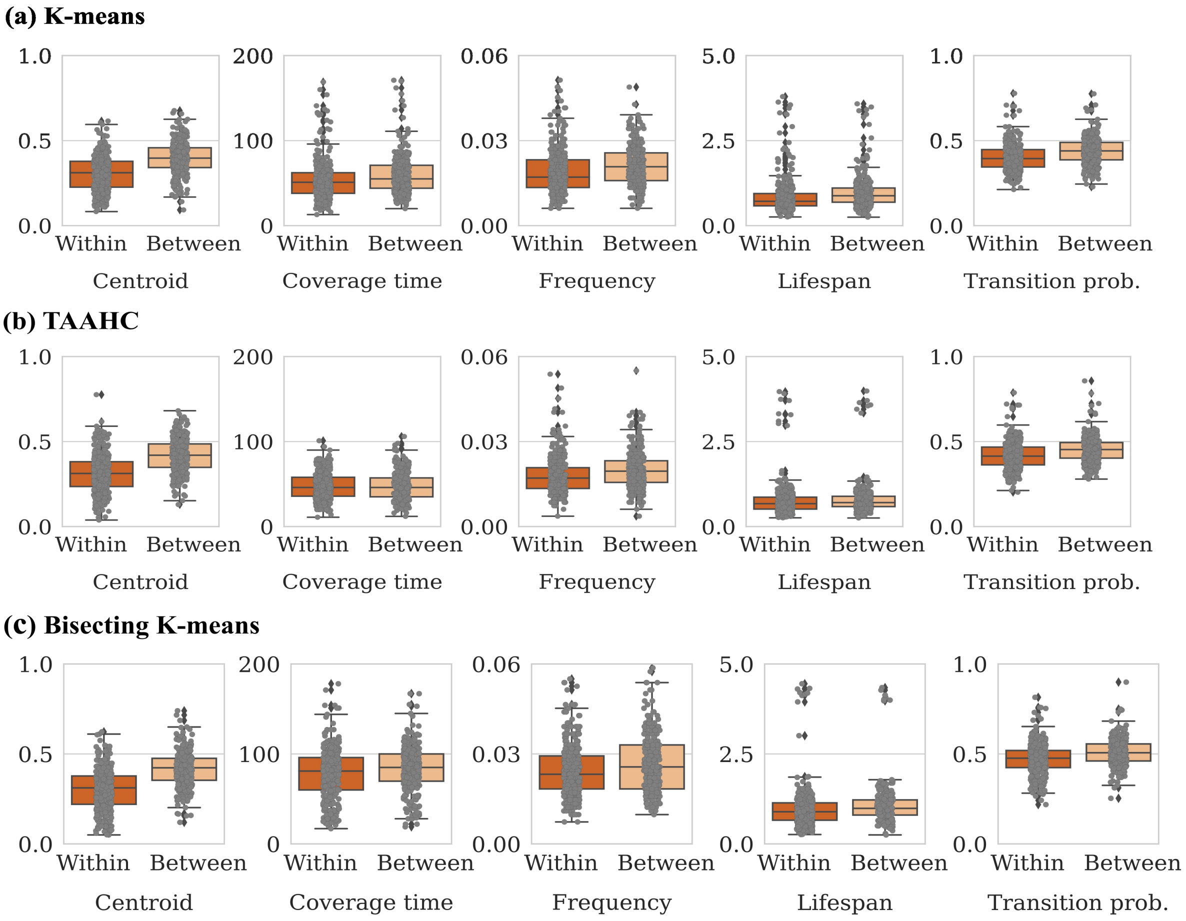

Figure S2: Within-participant and between-participant reproducibility of the state-transition dynamics with $K = 7$ states. (a) K-means. (b) TAAHC. (c) Bisecting K-means. "Within" and "Between" indicate the within-participant and between-participant comparisons, respectively. Each box plot shows the minimum, maximum, median, first quartile, and third quartile of the measurements. Each dot represents a session. "Centroid" abbreviates the centroid position, and "Transition prob." abbreviates the transition probability matrix.

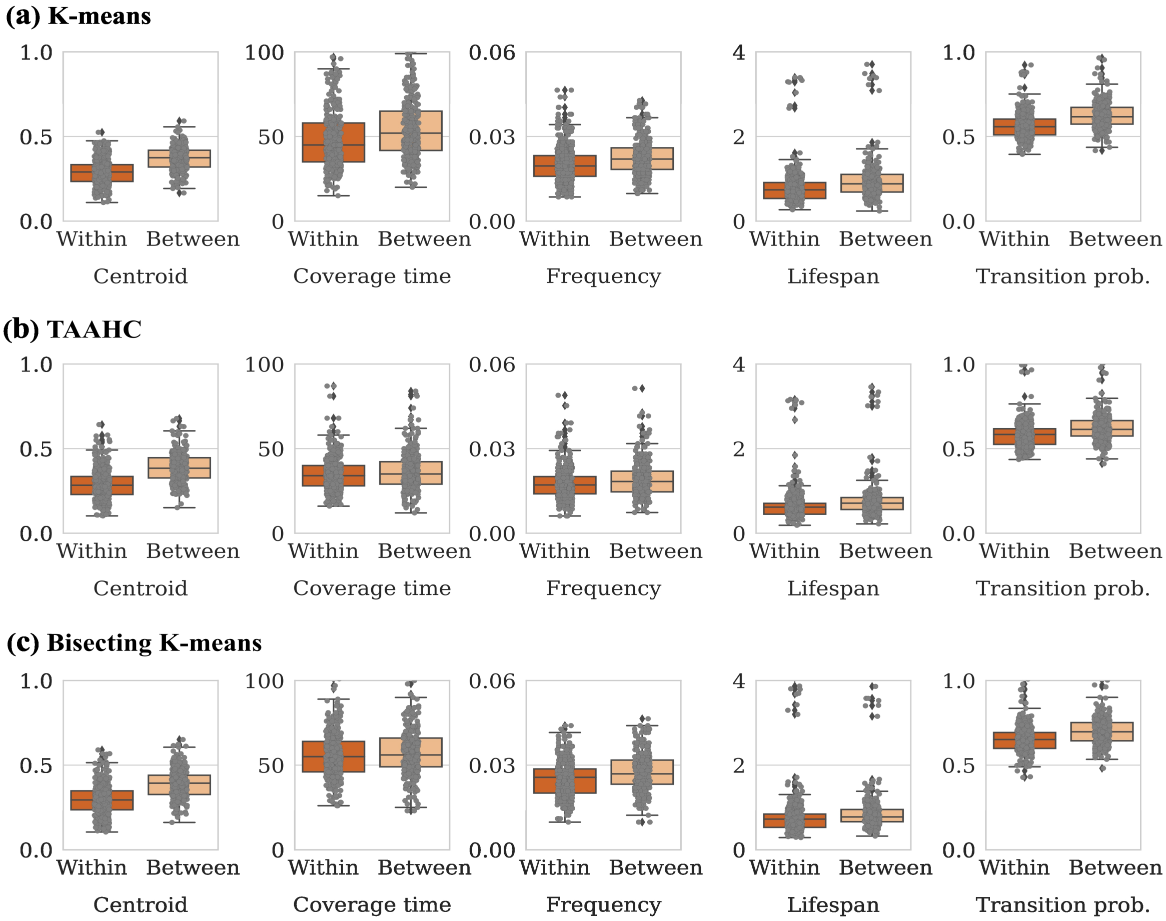

Figure S3: Within-participant and between-participant reproducibility of the state-transition dynamics with $K = 10$ states. (a) K-means. (b) TAAHC. (c) Bisecting K-means.

S4. Robustness test with the Euclidean distance used as dissimilarity between sessions

We mainly used the dissimilarity based on the $\text{corr}(\boldsymbol{c}_\ell, \boldsymbol{c}_{\ell'})$ defined by Eq. (7) in the main text to match and compare the set of the centroid positions, $\{\boldsymbol{c}_1, \ldots, \boldsymbol{c}_L\}$, between pairs of sessions. In this section, we investigate the robustness of the results of the test-retest reproducibility analysis by running the same analysis with the Euclidean distance between the centroid positions, defined by Eq. (14) in the main text, to match and compare the set of centroid positions between pairs of sessions. We also use the Euclidean distance to measure the discrepancy in terms of the centroid position between the two optimally matched sessions.

We show the p values for the permutation test for the three clustering methods, five discrepancy measures for the state-transition dynamics, and $K \in \{2, \ldots, 10\}$ in Tables S2, S3, and Table S4 for the DMN extracted from the MSC data, the whole-brain network extracted from the MSC data, and the whole-brain network extracted from the HCP data, respectively. In the tables, $p < 10^{-4}$ indicates that the deviation of the ND value from 1 is larger for the original session-to-session comparisons from 1 is larger than the deviation of the ND value for all the 10^4 randomized comparisons from 1. We find that the permutation test results obtained with the Euclidean distance are similar to those obtained with the cosine distance shown in the main text.

Quantitatively, for the DMN extracted from the MSC data, most p values (i.e., 128 out of the 135 comparisons; 94.81%) were smaller than 0.05 (indicated by *), and 111 of them (i.e., 83.07% of the 135 comparisons) remained significant after the Bonferroni correction (indicated by ***; equivalent to $p < 0.00037$, uncorrected). Moreover, we obtained $p < 0.001$, indicated with **, in 113 out of the 135 comparisons (i.e., 83.15%). We also found that the number of significant p values (with $p < 0.05$) for the K-means (45 out of 45) and bisecting K-means (44 out of 45), which differ only by 1 from each other, was somewhat larger than that for the TAAHC (42 out of 45). After the Bonferroni correction, the number of the significant p values remained larger for the K-means (43 out of 45) than for bisecting K-means (36 out of 45), which is larger than for the TAAHC (32 out of 45).

Table S2: Results of the permutation test for the DMN extracted from the MSC data when we used the Euclidean distance to calculate the distance between the centroids of clusters. *: $p < 0.05$, uncorrected; **: $p < 0.001$, uncorrected; ***: $p < 0.00037$, uncorrected (which is equivalent to $p < 0.05$, Bonferroni corrected). We remark that “Centroid” and ”Trans. prob.” abbreviate the centroid’s position and the transition probability matrix, respectively.

	K	Centroid	Coverage	Frequency	Lifespan	Trans. prob.
K-means	2	$< 10^{-4***}$	0.0186*	$< 10^{-4***}$	$< 10^{-4***}$	$< 10^{-4***}$
	3	$< 10^{-4***}$	$< 10^{-4***}$	$< 10^{-4***}$	$< 10^{-4***}$	$< 10^{-4***}$
	4	$< 10^{-4***}$	0.0014*	$< 10^{-4***}$	$< 10^{-4***}$	$< 10^{-4***}$
	5	$< 10^{-4***}$	$< 10^{-4***}$	$< 10^{-4***}$	$< 10^{-4***}$	$< 10^{-4***}$
	6	$< 10^{-4***}$	$< 10^{-4***}$	$< 10^{-4***}$	$< 10^{-4***}$	$< 10^{-4***}$
	7	$< 10^{-4***}$	$< 10^{-4***}$	$< 10^{-4***}$	$< 10^{-4***}$	$< 10^{-4***}$
	8	$< 10^{-4***}$	$< 10^{-4***}$	$< 10^{-4***}$	$< 10^{-4***}$	$< 10^{-4***}$
	9	$< 10^{-4***}$	$< 10^{-4***}$	$< 10^{-4***}$	$< 10^{-4***}$	$< 10^{-4***}$
	10	$< 10^{-4***}$	$< 10^{-4***}$	$< 10^{-4***}$	$< 10^{-4***}$	$< 10^{-4***}$
TAAHC	2	$< 10^{-4***}$	0.5528	0.0035*	0.0036*	0.002*
	3	$< 10^{-4***}$	0.1027	$< 10^{-4***}$	0.0026*	0.0001***
	4	$< 10^{-4***}$	0.0435*	$< 10^{-4***}$	$< 10^{-4***}$	$< 10^{-4***}$
	5	$< 10^{-4***}$	0.0842	0.0001***	$< 10^{-4***}$	$< 10^{-4***}$
	6	$< 10^{-4***}$	0.0076*	$< 10^{-4***}$	$< 10^{-4***}$	$< 10^{-4***}$
	7	$< 10^{-4***}$	0.5548	$< 10^{-4***}$	$< 10^{-4***}$	$< 10^{-4***}$
	8	$< 10^{-4***}$	0.0718	$< 10^{-4***}$	$< 10^{-4***}$	$< 10^{-4***}$
	9	$< 10^{-4***}$	0.0434*	$< 10^{-4***}$	$< 10^{-4***}$	$< 10^{-4***}$
	10	$< 10^{-4***}$	0.2352	$< 10^{-4***}$	$< 10^{-4***}$	$< 10^{-4***}$
Bisecting K-means	2	$< 10^{-4***}$	0.0287*	$< 10^{-4***}$	$< 10^{-4***}$	$< 10^{-4***}$
	3	$< 10^{-4***}$	0.0001***	$< 10^{-4***}$	$< 10^{-4***}$	$< 10^{-4***}$
	4	$< 10^{-4***}$	0.0584	$< 10^{-4***}$	$< 10^{-4***}$	$< 10^{-4***}$
	5	$< 10^{-4***}$	0.0419*	0.0003***	$< 10^{-4***}$	$< 10^{-4***}$
	6	$< 10^{-4***}$	0.018*	0.0001***	0.0004**	$< 10^{-4***}$
	7	$< 10^{-4***}$	0.0005**	$< 10^{-4***}$	$< 10^{-4***}$	$< 10^{-4***}$
	8	$< 10^{-4***}$	0.0037*	0.0002***	0.0001***	$< 10^{-4***}$
	9	$< 10^{-4***}$	0.0072*	0.0001***	$< 10^{-4***}$	$< 10^{-4***}$
	10	$< 10^{-4***}$	0.0067*	$< 10^{-4***}$	$< 10^{-4***}$	$< 10^{-4***}$

Table S3: Results of the permutation test for the whole-brain network extracted from the MSC data when we used the Euclidean distance to calculate the distance between the centroids of clusters. *: $p < 0.05$, uncorrected; **: $p < 0.001$, uncorrected; ***: $p < 0.00037$, uncorrected (which is equivalent to $p < 0.05$, Bonferroni corrected). We remark that "Centroid" and "Trans. prob." abbreviate the centroid's position and the transition probability matrix, respectively.

	K	Centroid	Coverage	Frequency	Lifespan	Trans. prob.
K-means	2	$< 10^{-4}$***	0.0368*	$< 10^{-4}$***	$< 10^{-4}$***	$< 10^{-4}$***
	3	$< 10^{-4}$***	0.0402*	$< 10^{-4}$***	$< 10^{-4}$***	$< 10^{-4}$***
	4	$< 10^{-4}$***	0.0353*	$< 10^{-4}$***	$< 10^{-4}$***	$< 10^{-4}$***
	5	$< 10^{-4}$***	$< 10^{-4}$***	$< 10^{-4}$***	$< 10^{-4}$***	$< 10^{-4}$***
	6	$< 10^{-4}$***	0.0007**	$< 10^{-4}$***	$< 10^{-4}$***	$< 10^{-4}$***
	7	$< 10^{-4}$***	0.0016*	$< 10^{-4}$***	$< 10^{-4}$***	$< 10^{-4}$***
	8	$< 10^{-4}$***	0.0004**	$< 10^{-4}$***	$< 10^{-4}$***	$< 10^{-4}$***
	9	$< 10^{-4}$***	10^{-4}***	$< 10^{-4}$***	$< 10^{-4}$***	$< 10^{-4}$***
	10	$< 10^{-4}$***	0.0003***	$< 10^{-4}$***	$< 10^{-4}$***	$< 10^{-4}$***
TAAHC	2	$< 10^{-4}$***	0.0776	0.0001***	$< 10^{-4}$***	0.0001***
	3	$< 10^{-4}$***	0.0463*	$< 10^{-4}$***	0.0003***	$< 10^{-4}$***
	4	$< 10^{-4}$***	0.0940	$< 10^{-4}$***	0.0011*	0.0004**
	5	$< 10^{-4}$***	0.0813	$< 10^{-4}$***	$< 10^{-4}$***	$< 10^{-4}$***
	6	$< 10^{-4}$***	0.7868	$< 10^{-4}$***	0.0011*	$< 10^{-4}$***
	7	$< 10^{-4}$***	0.8686	$< 10^{-4}$***	$< 10^{-4}$***	$< 10^{-4}$***
	8	$< 10^{-4}$***	0.0875	$< 10^{-4}$***	$< 10^{-4}$***	$< 10^{-4}$***
	9	$< 10^{-4}$***	0.0403*	$< 10^{-4}$***	$< 10^{-4}$***	$< 10^{-4}$***
	10	$< 10^{-4}$***	0.036*	$< 10^{-4}$***	$< 10^{-4}$***	$< 10^{-4}$***
Bisecting K-means	2	$< 10^{-4}$***	0.0303*	$< 10^{-4}$***	$< 10^{-4}$***	$< 10^{-4}$***
	3	$< 10^{-4}$***	0.2482	$< 10^{-4}$***	0.0041*	0.0174*
	4	$< 10^{-4}$***	0.0576	$< 10^{-4}$***	$< 10^{-4}$***	$< 10^{-4}$***
	5	$< 10^{-4}$***	0.2599	0.0002***	0.0003***	0.0483*
	6	$< 10^{-4}$***	0.0658	$< 10^{-4}$***	$< 10^{-4}$***	$< 10^{-4}$***
	7	$< 10^{-4}$***	0.6427	0.0002***	$< 10^{-4}$***	0.0001***
	8	$< 10^{-4}$***	$< 10^{-4}$***	$< 10^{-4}$***	$< 10^{-4}$***	$< 10^{-4}$***
	9	$< 10^{-4}$***	0.0905	0.0018*	$< 10^{-4}$***	$< 10^{-4}$***
	10	$< 10^{-4}$***	0.0031*	$< 10^{-4}$***	$< 10^{-4}$***	$< 10^{-4}$***

Table S4: Results of the permutation test for the whole-brain network extracted from the HCP data when we used the Euclidean distance to calculate the distance between the centroids of clusters. *: $p < 0.05$, uncorrected; **: $p < 0.001$, uncorrected; ***: $p < 0.00037$, uncorrected (which is equivalent to $p < 0.05$, Bonferroni corrected). We remark that "Centroid" and "Trans. prob." abbreviate the centroid's position and the transition probability matrix, respectively.

	K	Centroid	Coverage	Frequency	Lifespan	Trans. prob.
K-means	2	$< 10^{-4***}$	0.0023*	$< 10^{-4***}$	$< 10^{-4***}$	$< 10^{-4***}$
	3	$< 10^{-4***}$	$< 10^{-4***}$	$< 10^{-4***}$	$< 10^{-4***}$	$< 10^{-4***}$
	4	$< 10^{-4***}$	$< 10^{-4***}$	$< 10^{-4***}$	$< 10^{-4***}$	$< 10^{-4***}$
	5	$< 10^{-4***}$	$< 10^{-4***}$	$< 10^{-4***}$	$< 10^{-4***}$	$< 10^{-4***}$
	6	$< 10^{-4***}$	$< 10^{-4***}$	$< 10^{-4***}$	$< 10^{-4***}$	$< 10^{-4***}$
	7	$< 10^{-4***}$	$< 10^{-4***}$	$< 10^{-4***}$	$< 10^{-4***}$	$< 10^{-4***}$
	8	$< 10^{-4***}$	$< 10^{-4***}$	$< 10^{-4***}$	$< 10^{-4***}$	$< 10^{-4***}$
	9	$< 10^{-4***}$	$< 10^{-4***}$	$< 10^{-4***}$	$< 10^{-4***}$	$< 10^{-4***}$
	10	$< 10^{-4***}$	$< 10^{-4***}$	$< 10^{-4***}$	$< 10^{-4***}$	$< 10^{-4***}$
TAAHC	2	$< 10^{-4***}$	$< 10^{-4***}$	$< 10^{-4***}$	$< 10^{-4***}$	$< 10^{-4***}$
	3	$< 10^{-4***}$	0.0952	$< 10^{-4***}$	$< 10^{-4***}$	$< 10^{-4***}$
	4	$< 10^{-4***}$	0.0159*	$< 10^{-4***}$	$< 10^{-4***}$	$< 10^{-4***}$
	5	$< 10^{-4***}$	0.1318	$< 10^{-4***}$	$< 10^{-4***}$	$< 10^{-4***}$
	6	$< 10^{-4***}$	0.0191*	$< 10^{-4***}$	$< 10^{-4***}$	$< 10^{-4***}$
	7	$< 10^{-4***}$	0.0096*	$< 10^{-4***}$	$< 10^{-4***}$	$< 10^{-4***}$
	8	$< 10^{-4***}$	0.0007**	$< 10^{-4***}$	$< 10^{-4***}$	$< 10^{-4***}$
	9	$< 10^{-4***}$	0.0003***	$< 10^{-4***}$	$< 10^{-4***}$	$< 10^{-4***}$
	10	$< 10^{-4***}$	0.0008**	$< 10^{-4***}$	$< 10^{-4***}$	$< 10^{-4***}$
Bisecting K-means	2	$< 10^{-4***}$	0.0016*	$< 10^{-4***}$	$< 10^{-4***}$	$< 10^{-4***}$
	3	$< 10^{-4***}$	0.013*	$< 10^{-4***}$	$< 10^{-4***}$	$< 10^{-4***}$
	4	$< 10^{-4***}$	$< 10^{-4***}$	$< 10^{-4***}$	$< 10^{-4***}$	$< 10^{-4***}$
	5	$< 10^{-4***}$	$< 10^{-4***}$	$< 10^{-4***}$	$< 10^{-4***}$	$< 10^{-4***}$
	6	$< 10^{-4***}$	0.0001***	$< 10^{-4***}$	$< 10^{-4***}$	$< 10^{-4***}$
	7	$< 10^{-4***}$	0.0118*	$< 10^{-4***}$	$< 10^{-4***}$	$< 10^{-4***}$
	8	$< 10^{-4***}$	$< 10^{-4***}$	$< 10^{-4***}$	$< 10^{-4***}$	$< 10^{-4***}$
	9	$< 10^{-4***}$	$< 10^{-4***}$	$< 10^{-4***}$	$< 10^{-4***}$	$< 10^{-4***}$
	10	$< 10^{-4***}$	$< 10^{-4***}$	$< 10^{-4***}$	$< 10^{-4***}$	$< 10^{-4***}$

S5. Impact of the number of ROIs in the DMN on test-retest reproducibility

In this section, we explore the influence of the number of ROIs given by different coordinate systems on test-retest reproducibility. To this end, we use the DMN with 59 ROIs extracted from MSC dataset that is part of the 264-ROI system that we have used for constructing the whole-brain network [1]. We followed the same analysis pipeline employed in the main text. For the 59-ROI DMN, we solely removed the global signal without averaging the time series of each left- and right-hemispheric pair of symmetric ROIs.

We show the p values for the permutation test across three clustering methods, five discrepancy measures for the state-transition dynamics, and $K \in \{2, \cdots, 10\}$ in Table S5. We find the within-participant reproducibility relative to the between-participant reproducibility is similarly high with the present 59-ROI DMN compared to the case of the 12-ROI DMN (see Table 2 in the main text), except that the coverage in the case of K-means yields poor results for the 59-ROI DMN but not for the 12-ROI DMN.

Table S5: Results of the permutation test for the 59-ROI DMN extracted from the MSC data. *: $p < 0.05$, uncorrected; **: $p < 0.001$, uncorrected; ***: $p < 0.00037$ uncorrected (which is equivalent to $p < 0.05$, Bonferroni corrected). We remark that “Centroid” and ”Trans. prob.” abbreviate the centroid’s position and the transition probability matrix, respectively.

	K	Centroid	Coverage	Frequency	Lifespan	Trans. prob.
K-means	2	$< 10^{-4***}$	0.0285*	$< 10^{-4***}$	0.0002***	$< 10^{-4***}$
	3	$< 10^{-4***}$	0.5994	$< 10^{-4***}$	$< 10^{-4***}$	$< 10^{-4***}$
	4	$< 10^{-4***}$	0.5626	$< 10^{-4***}$	0.0001***	$< 10^{-4***}$
	5	$< 10^{-4***}$	0.7903	$< 10^{-4***}$	$< 10^{-4***}$	$< 10^{-4***}$
	6	$< 10^{-4***}$	0.0004**	$< 10^{-4***}$	$< 10^{-4***}$	$< 10^{-4***}$
	7	$< 10^{-4***}$	0.0232*	$< 10^{-4***}$	$< 10^{-4***}$	$< 10^{-4***}$
	8	$< 10^{-4***}$	0.1162	$< 10^{-4***}$	$< 10^{-4***}$	$< 10^{-4***}$
	9	$< 10^{-4***}$	0.2576	$< 10^{-4***}$	$< 10^{-4***}$	$< 10^{-4***}$
	10	$< 10^{-4***}$	0.2229	$< 10^{-4***}$	$< 10^{-4***}$	$< 10^{-4***}$
TAAHC	2	$< 10^{-4***}$	0.3201	$< 10^{-4***}$	$< 10^{-4***}$	$< 10^{-4***}$
	3	$< 10^{-4***}$	0.4577	$< 10^{-4***}$	0.0001***	$< 10^{-4***}$
	4	$< 10^{-4***}$	0.1850	$< 10^{-4***}$	$< 10^{-4***}$	$< 10^{-4***}$
	5	$< 10^{-4***}$	0.3747	$< 10^{-4***}$	$< 10^{-4***}$	$< 10^{-4***}$
	6	$< 10^{-4***}$	0.4879	$< 10^{-4***}$	$< 10^{-4***}$	$< 10^{-4***}$
	7	$< 10^{-4***}$	0.3990	$< 10^{-4***}$	$< 10^{-4***}$	$< 10^{-4***}$
	8	$< 10^{-4***}$	0.5510	$< 10^{-4***}$	$< 10^{-4***}$	$< 10^{-4***}$
	9	$< 10^{-4***}$	0.6263	$< 10^{-4***}$	$< 10^{-4***}$	$< 10^{-4***}$
	10	$< 10^{-4***}$	0.7597	$< 10^{-4***}$	$< 10^{-4***}$	$< 10^{-4***}$
Bisecting K-means	2	$< 10^{-4***}$	0.0184*	$< 10^{-4***}$	$< 10^{-4***}$	$< 10^{-4***}$
	3	$< 10^{-4***}$	0.0311*	$< 10^{-4***}$	$< 10^{-4***}$	$< 10^{-4***}$
	4	$< 10^{-4***}$	0.7056	$< 10^{-4***}$	0.0002***	$< 10^{-4***}$
	5	$< 10^{-4***}$	0.1006	$< 10^{-4***}$	$< 10^{-4***}$	$< 10^{-4***}$
	6	$< 10^{-4***}$	0.5694	$< 10^{-4***}$	0.0024*	$< 10^{-4***}$
	7	$< 10^{-4***}$	0.0343*	$< 10^{-4***}$	$< 10^{-4***}$	$< 10^{-4***}$
	8	$< 10^{-4***}$	0.0006**	$< 10^{-4***}$	$< 10^{-4***}$	$< 10^{-4***}$
	9	$< 10^{-4***}$	0.0053*	$< 10^{-4***}$	0.0001***	$< 10^{-4***}$
	10	$< 10^{-4***}$	0.2402	< 0.0001***	0.0006**	$< 10^{-4***}$

References

[1] Power JD, Cohen AL, Nelson SM, Wig GS, Barnes KA, Church JA, Vogel AC, Laumann TO, Miezin FM, Schlaggar BL, et al. Functional network organization of the human brain. *Neuron*, 72:665–678, 2011.
